# Stochastic Gauss-Newton method for estimating absorption and scattering in optical tomography with the Monte Carlo method for light transport

**DOI:** 10.1364/BOE.528666

**Published:** 2024-07-30

**Authors:** Jonna Kangasniemi, Meghdoot Mozumder, Aki Pulkkinen, Tanja Tarvainen

**Affiliations:** University of Eastern Finland, Department of Technical Physics, P.O. Box 1627, 70211 Kuopio, Finland

## Abstract

Image reconstruction in optical tomography in the so-called transport regime, where the diffusion approximation is not valid, requires modeling of light transport using the radiative transfer equation. In this work, we approach this problem by utilizing the Monte Carlo method for light transport. In this work, we propose a methodology for absolute imaging of absorption and scattering in this regime utilizing a Monte Carlo method for light transport. The image reconstruction problem is formulated as a minimization problem that is solved using a stochastic Gauss-Newton method. In the construction of the Jacobian matrix for scattering, a perturbation approximation for Monte Carlo is utilized. The approach is evaluated with numerical simulations using an adaptive approach where the number of photon packets is adjusted during the iterations, and with different fixed numbers of photon packets. The simulations show that the Monte Carlo method for light transport can be utilized in the absolute imaging problem of optical tomography and that the absorption and scattering parameters can be estimated simultaneously with good accuracy.

## 1. Introduction

Optical imaging is a non-invasive imaging technique that uses visible or near-infrared light to interrogate the internal properties of biological tissues [1,2]. In optical tomography (OT), the aim is to form three-dimensional images of the optical properties of the target from light transport measurements made on the boundary of the target [2,3]. Applications of OT are, for example, in monitoring treatments [2], imaging breast cancer [4], diagnosis of rheumatoid arthritis [5,6], functional brain studies, monitoring infant brain oxygenation level, and small animal studies [4,7,8].

The image reconstruction problem in OT is a highly ill-posed problem, and therefore it needs to be approached in the framework of inverse problems [2,3,9]. In the numerical solution of this problem, modeling light propagation in the imaging situation is needed. Furthermore, solving the inverse problem is often formulated as a minimization problem, which solution requires evaluation of the gradients or Jacobian of the light transport model. In OT, the inverse problem is often considered either as a difference imaging problem or an absolute imaging problem. In difference imaging, changes in optical parameters between two measurement times are reconstructed. In absolute imaging, on the other hand, the absolute absorption and scattering values are estimated using a single set of measurements. In this work, the absolute image reconstruction problem for determining absorption and scattering distributions in OT is considered.

Light propagation in biological tissues can be modeled with the radiative transfer equation (RTE). Solving the inverse problem of OT with the RTE as a light transport model has been studied for example in [10–13]. However, due to the computational complexity of the RTE, the DA is generally utilized in optical imaging. The DA is a valid approximation in a highly scattering medium, relatively far (longer than a few scattering lengths) from the source. Therefore, if the imaged target is small or contains low-scattering regions, light propagation needs to be modeled using the RTE or other approximations [2,3,14].

As an alternative to deterministic models, the Monte Carlo method can be used to simulate light propagation in tissue. The Monte Carlo method for light transport is a stochastic method in which light transport is approximated by simulating a large number of photons or photon packets and tracing their paths in a medium [15]. The method is widely accepted as an accurate approach to simulate the light transport in biological tissues, and it has been utilized in various studies, for more information, see e.g. [16–20].

In this work, the use of the Monte Carlo method for light transport in the absolute image reconstruction problem of OT is proposed. Monte Carlo method has previously been used in OT in difference imaging of either absorption or scattering [21–23] or both [24,25]. Further, difference imaging of blood volume, oxygen saturation, and water content was studied in [26], and in [25], absolute imaging of absorption and scattering was studied utilizing a difference approximation for Jacobian. Furthermore, in addition to OT, the methodology has been utilized in the inverse problem of quantitative photoacoustic tomography and ultrasound-modulated diffuse optical tomography [27–30]. Outside optical imaging, a methodology where a Markov Chain Monte Carlo method is utilized in approximating the posterior distribution with a Monte Carlo forward model was presented in [31] with an application in atmospheric remote sensing.

In this work, absolute imaging of absorption and scattering coefficients is proposed utilizing a photon packet Monte Carlo method for light transport. The image reconstruction problem is formulated as a minimization problem that is solved using a Gauss-Newton method. A so-called perturbation Monte Carlo is utilized in the evaluation of the Jacobian matrix for scattering. A similar approach has been utilized in quantitative photoacoustic tomography for example in [28,30]. Furthermore, we utilize an adaptive stochastic Gauss-Newton method [27,29,30] for controlling the amount of photon packets during the Gauss-Newton iteration. This enables adjusting the amount of photon packets with respect to the stochastic noise, leading to efficient usage of computational resources during iteration.

The rest of the paper is organized as follows. In Sec. 2, the image reconstruction problem of OT is reviewed together with a review of the Monte Carlo method for light transport. Further, a stochastic Gauss-Newton method and an adaptive approach for solving the OT image reconstruction problem are presented. The proposed approach is evaluated with numerical simulations in Sec. 3. Finally, the results are discussed and conclusions are given in Sec. 4.

## 2. Methods

In OT experiments, the imaged target is illuminated from different locations at its boundary, and the amount of transmitted light on the boundary is measured using light-sensitive detectors [2]. The measurement systems are based either on continuous-wave, time-domain, or frequency-domain implementation [1]. In this work, we consider the frequency-domain setup where the input light is intensity modulated by electromagnetic wave, and the amplitude and phase delay of the transmitted signal are measured. In the inverse problem of OT, images of absorption and scattering are reconstructed from the measured data [3]. In this work, a Bayesian approach to this image reconstruction problem is taken. Thus, all parameters are considered as random variables, and a *maximum a posteriori* estimate is sought by minimizing [9]

(1) $$\underset{x}{\arg\;\min}\;\{u(x)\}=\underset{x}{\arg\;\min}\;\left\{\frac{1}{2}{\left\|L_{e}(Y_{meas}-Y(x)-\eta_{e})\right\|}^{2}+\frac{1}{2}{\left\|L_{x}(x-\eta_{x})\right\|}^{2}\right\},$$

where $Y_{meas}=(Y_{meas,1},\ldots ,Y_{meas,M})\in R^{M}$ is a vector of measurements, $Y\;:\;R^{2K}\to R^{M}$ is a discretized forward model that maps the absorption and scattering parameters to the measurable data, $x={\left(\mu_{a},\;\mu_{s}\right)}^{T}$ , where $\mu_{a}=(\mu_{a,1},\ldots ,\mu_{a,K})\in R^{K}$ and $\mu_{s}=(\mu_{s,1},\ldots ,\mu_{s,K})\in R^{K}$ are discretized absorption and scattering coefficients. Further, $\eta_{e}$ and $L_{e}$ are the expected value and Cholesky decomposition of the inverse of the covariance matrix $\Gamma_{e}$ of the noise *e*, where $\Gamma_{e}^{-1}=L_{e}^{T}L_{e}$ , and $\eta_{x}$ and $L_{x}$ are the expected value and Cholesky decomposition of the inverse of the covariance matrix $\Gamma_{x}$ of the unknown parameters *x*, where $\Gamma_{x}^{-1}=L_{x}^{T}L_{x}$ . Formulation (1) can also be interpreted in the regularization framework, where the first term of the functional u(x) corresponds to a (weighted) least-squares minimization between data and model predictions, and the second term is a regularizing penalty functional.

The minimization problem (1) can be solved using methods of computational optimization, such as the Gauss-Newton method. In the Gauss-Newton method, the estimates $x_{i}$ are updated such that

(2) $$x_{i+1}=x_{i}+\alpha_{i}\delta (x_{i}),$$

where $\alpha_{i}$ is the step length parameter and $\delta (x_{i})$ is the Gauss-Newton minimization direction on iteration *i*. The minimization direction can be solved from

(3) $$\left(J^{T}(x_{i})\Gamma_{e}^{-1}J(x_{i})+\Gamma_{x}^{-1}\right)\delta (x_{i})=J^{T}(x_{i})\Gamma_{e}^{-1}\left(Y_{meas}-Y(x_{i})-\eta_{e}\right)-\Gamma_{x}^{-1}\left(x_{i}-\eta_{x}\right),$$

where $J(x_{i})=\left(J_{\mu_{a}}(x_{i})\;,\;J_{\mu_{s}}(x_{i})\right)$ is the Jacobian of the forward model.

In this work, the solution of the forward model is based on a numerical approximation of the RTE with the Monte Carlo method. In the frequency domain, the RTE is of the form [3,14,32]

(4) $$\left\{ \begin{array}{l} \begin{array}{l} \left(\frac{i\omega }{c}+\hat{s}\cdot \nabla +\mu_{s}(r)+\mu_{a}(r)\right)\phi (r,\hat{s})=\mu_{s}(r)\int_{Sd-1}\Theta (\hat{s}\cdot \hat{s}\prime )\phi (r,\hat{s}\prime )d\hat{s}\prime ,\;r\in \Omega \end{array}\\ \phi (r,\hat{s})=\left\{ \begin{array}{ll} \phi_{0}(r,\hat{s}), & r\in \cup_{j}\epsilon_{j},\;\hat{s}\cdot \hat{n}<0\\ 0, & r\in \partial \Omega \setminus \cup_{j}\epsilon_{j},\;\hat{s}\cdot \hat{n}<0\; \end{array}\right. \end{array}\right.$$

where $\Omega \subset R^{d}$ is a domain with dimensions d=2 or 3 and a boundary $\partial \Omega ,\hat{s}\in S^{d-1}$ is a unit vector in the direction of interest, i is the imaginary unit, ω=2πf is the angular modulation frequency of the input signal and *f* is the modulation frequency, $c=c_{0}/n$ is the speed of light in the medium with *n* being the refractive index and $c_{0}$ the speed of light in a vacuum. Further, $\mu_{a}(r)$ is the absorption coefficient, $\mu_{s}(r)$ is the scattering coefficients, $\phi (r,\hat{s})$ is the radiance, $\phi_{0}(r,\hat{s})$ is a boundary source at a position $\epsilon_{j},\hat{n}$ is an outward unit normal on the boundary, and $\Theta (\hat{s}\cdot \hat{s}\prime )$ is the scattering phase function. In OT, a commonly used phase function is the Henyey-Greenstein scattering function [33]

(5) $$\Theta (\hat{s}\cdot \hat{s}\prime )=\left\{ \begin{array}{ll} \frac{1}{2\pi }\frac{1-g^{2}}{1+g^{2}-2g-2\hat{s}\cdot \hat{s}\prime } & d=2\\ \frac{1}{4\pi }\frac{1-g^{2}}{{\left(1+g^{2}-2g-2\hat{s}\cdot \hat{s}\prime \right)}^{3/2}} & d=3, \end{array}\right.$$

where −1<g<1 is a scattering anisotropy parameter. The boundary condition above assumes that, in the boundary, no photons travel in inward directions except at the source locations $\epsilon_{j}\subset \delta \Omega$ . The measurable quantity in OT is the exitance Y on the boundary of the domain, which is defined as

(6) $$Y(r)=\int_{S^{d-1}}\phi (r,\hat{s})(\hat{s}\cdot \hat{n})d\hat{s}.$$

### 2.1 Monte Carlo method for light transport

The solution of the RTE (4) can be approximated using the Monte Carlo method for light transport. In the photon packet Monte Carlo [15], packets of photons are simulated with an initial weight $w_{0}$ from the source locations. Then, the photon packets propagate within the domain while undergoing scattering events and a decreasing weight [15,17,34]. Let $\mu_{a}ds$ be the probability for a photon absorption in a small length ds in the propagation direction. Similarly, let $\mu_{s}ds$ be the probability for a photon scattering. The probability for absorption follows an exponential probability density function. Absorption is taken into account by reducing the weight along the trajectory *s* of the photon packet

(7) $$w(s)=w_{0}\exp\left(-\int_{0}^{s}\mu_{a}(s\prime )+\frac{i\omega }{c}ds\prime \right).$$

The scattering length *l* is the path length that the photon travels before scattering. It follows an exponential probability density function

(8) $$f(l)=\mu_{s}(l)\exp\left(-\int_{0}^{l}\mu_{s}(l\prime )dl\prime \right).$$

The scattering direction of the scattering event is based on the probability distribution of the scattering angle, which in this work is the Henyey-Greenstein phase function (5). In the Monte Carlo simulation, propagation of a photon packet is followed until the photon packet exits the simulation domain or its weight is small enough to be dismissed [15].

At the end of the simulation, the exitance (6) on a boundary element *b* of is obtained from

(9) $$\begin{array}{ll} Y_{b} & =\frac{W_{b}}{PA_{b}}=\frac{1}{PA_{b}}\sum_{phot}w_{phot}(s)\\ & =\frac{1}{PA_{b}}\sum_{phot}w_{0}\exp\left(-\sum_{h}\left(\mu_{a,k}+\frac{i\omega }{c}\right)l_{h,phot}\right) \end{array}$$

where $W_{b}$ is the weight of the photon packets that escaped through the boundary element *b*, *P* is the number of photon packets, $A_{b}$ is the length (d=2) or area (d=3) of the boundary element, and "phot" refers to the photon packets that escaped through boundary element *b* [21,34]. Further, $l_{h,phot}$ is the photon path length in a discretization element *h* of piece-wise constant discretized optical parameters.

### 2.2 Stochastic Gauss-Newton method

In this work, to consider the stochastic nature of the Monte Carlo, the minimization problem (1) is approached using methods of stochastic optimization [27,35]. Following the approach presented in [27,29,30], the stochastic Gauss-Newton (SGN) method can be derived. In the SGN, estimates $x_{i}$ are updated such that

(10) $$x_{i+1}=x_{i}+\alpha_{i}\delta_{P_{i}}(x_{i}),$$

where $\alpha_{i}$ is the step length parameter and $\delta_{P_{i}}(x_{i})$ is the approximate Gauss-Newton minimization direction calculated with $P_{i}$ photon packets. The minimization direction can be solved from

(11) $$\left(J_{P_{i}}^{T}(x_{i})\Gamma_{e}^{-1}J_{P_{i}}(x_{i})+\Gamma_{x}^{-1}\right)\delta_{P_{i}}(x_{i})=J_{P_{i}}^{T}(x_{i})\Gamma_{e}^{-1}\left(Y_{meas}-Y_{P_{i}}(x_{i})-\eta_{e}\right)-\Gamma_{x}^{-1}\left(x_{i}-\eta_{x}\right),$$

where $Y_{P_{i}}$ is the stochastic forward model and $J_{P_{i}}$ is the stochastic Jacobian evaluated at point $x_{i}$ with *P* photon packets on iteration *i*. In this work, Eq. 11 was solved using a MATLAB built-in function mldivide.

The Jacobian matrix is constructed from derivatives of the exitance with respect to the absorption and scattering coefficients. The derivative with respect to absorption can be calculated directly from Eqs. (6) and (9) [21,22]. The derivative of exitance $Y_{b}$ on a boundary element *b* with respect to absorption coefficient $\mu_{a,k}$ in a discretization element *k* is

(12) $$\begin{array}{ll} \frac{\partial Y_{b}}{\partial \mu_{a,k}} & =\frac{1}{PA_{b}}\sum_{phot}-l_{k,phot}w_{phot}(s)\\ & =\frac{1}{PA_{b}}\sum_{phot}-l_{k,phot}w_{0}\exp\left(-\sum_{h}\left(\mu_{a,h}+\frac{i\omega }{c}\right)l_{h,phot}\right). \end{array}$$

To obtain the derivatives for scattering, a so-called perturbation Monte Carlo method is utilized [22]. In the perturbation Monte Carlo, the effect of a small change in the optical parameters (perturbation) on the simulations is evaluated by reusing the trajectories from unperturbed simulations [28]. Then, the perturbed weight $\tilde{w}$ of a perturbed scattering coefficient ${\tilde{\mu }}_{s}$ can be derived

(13) $$\tilde{w}=w{\left(\frac{{\tilde{\mu }}_{s}}{\mu_{s}}\right)}^{n_{s}}\exp\left(-({\tilde{\mu }}_{s}-\mu_{s})L_{tot}\right),$$

where *w* is the unperturbed weight, $n_{s}$ is the number of scattering events in the perturbed region, and $L_{tot}$ is the total distance that the photon packet has traveled inside the perturbed region [22,28]. Then, the derivative of exitance $Y_{b}$ on a boundary element *b* with respect to scattering coefficient $\mu_{s,k}$ in a discretization element *k* can be expressed as

(14) $$\begin{array}{ll} \frac{\partial Y_{b}}{\partial \mu_{s,k}} & =\frac{1}{PA_{b}}\sum_{phot}\left(\frac{n_{s}}{\mu_{s,k}}-l_{k,phot}\right)w_{phot}(s)\\ & =\frac{1}{PA_{b}}\sum_{phot}\left(\frac{n_{s}}{\mu_{s,k}}-l_{k,phot}\right)w_{0}\exp\left(-\sum_{h}\left(\mu_{a,h}+\frac{i\omega }{c}\right)l_{h,phot}\right). \end{array}$$

### 2.3 Adaptive stochastic Gauss-Newton method

In this work, the adaptive stochastic Gauss-Newton method (ASGN) is used to control the number of photon packets on each Gauss-Newton iteration [29]. The methodology uses a low number of photon packets when the minimized functional is large, and increases this number based on the amount of stochastic noise in simulations as iteration proceeds and the estimates approach the minimum [27,29,30].

In the ASGN method, *L* samples of the approximate forward solutions $\left\{Y_{P_{i}}^{\left(l\right)}(x_{i})\right\}$ and Jacobians $\left\{J_{P_{i}}^{\left(l\right)}(x_{i})\right\}$ are calculated using $P_{i}$ photon packets, for ℓ=1,…,L on each iteration. These samples are used to calculate a set of minimization directions $\left\{\delta_{P_{i}}^{\left(l\right)}(x_{i})\right\}$ . Furthermore, approximations of the accurate solution $Y(x_{i})$ and its Jacobian $J(x_{i})$ are calculated by taking averages of the samples. These are used to calculate an approximation of an ’accurate’ Gauss-Newton minimization direction $\delta (x_{i})$ . The calculated minimization directions $\left\{\delta_{P_{i}}^{\left(l\right)}(x_{i})\right\}$ and the approximation of the accurate minimization direction $\delta (x_{i})$ are used to evaluate the accuracy of the minimization direction using a norm test [27,29]. The norm test for the ASGN method is

(15) $$V_{P_{i}}{\left(x_{i}\right)}^{2}:=\frac{E\left\{{\left\|\delta (x_{i})-\delta_{P_{i}}(x_{i})\right\|}^{2}\right\}}{{\left\|\delta (x_{i})\right\|}^{2}}\leq \gamma^{2},\;\gamma >0,$$

where $V_{P_{i}}{\left(x_{i}\right)}^{2}$ describes the expected value of the squared relative error evaluated at a point $x_{i}$ with $P_{i}$ photon packets and *γ* is a threshold parameter which defines the acceptable relative error in the minimization direction [29,35,36]. If the norm test fails, the error in the minimization direction is considered to be too large and the number of photon packets is increased by a factor

(16) $$P_{i}\leftarrow \frac{V_{P}{\left(x_{i}\right)}^{2}}{\gamma^{2}}P_{i}.$$

The ASGN algorithm for OT is presented in Algorithm 1.

## 3. Simulation studies

**Fig. 1.**
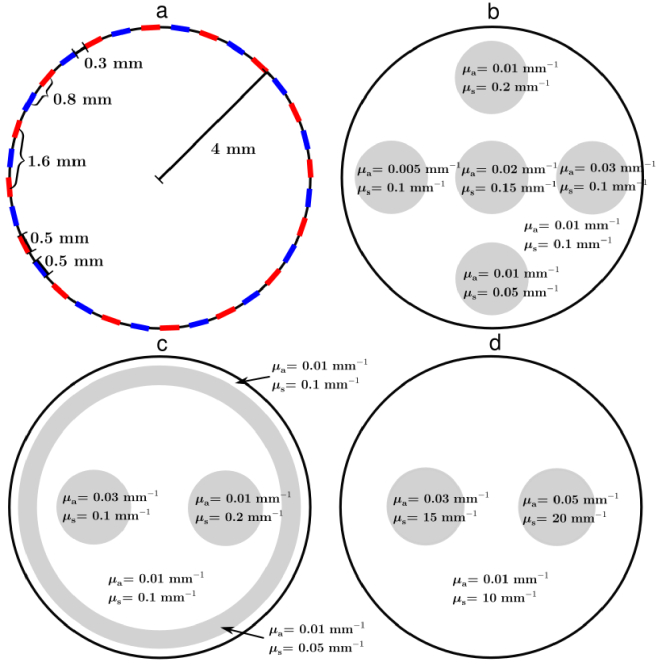
a) Illustration of the simulation domain with the locations of the sources (red line) and detectors (blue line) indicated. Illustrations of the simulation targets: b) the target with absorbing and scattering circular inclusions, c) the target with an absorbing and a scattering inclusion inside a low-scattering layer, and d) the highly scattering target.

The proposed methodology was evaluated with two-dimensional numerical simulations. A circular domain with a radius of 4 mm was considered. The simulation setup consisted of 16 sources and 16 detectors with a width of 0.5 mm, equally distributed on the boundary of the domain. Thus, the shortest source-detector distance in the simulation setup was 0.8 mm. The simulation domain and the locations of the sources and detectors are illustrated in Fig. 1 a). The shape of the light sources in the Monte Carlo implementation was modeled as spatially uniform and angularly cosine, which means that the initial directions of the photon packets followed a cosine distribution. The simulations were done in MATLAB (R2020b, MathWorks Inc., Natick, Massachusetts, United States), and the Monte Carlo simulations were implemented in C++.

**Algorithm 1.** Adaptive stochastic Gauss-Newton

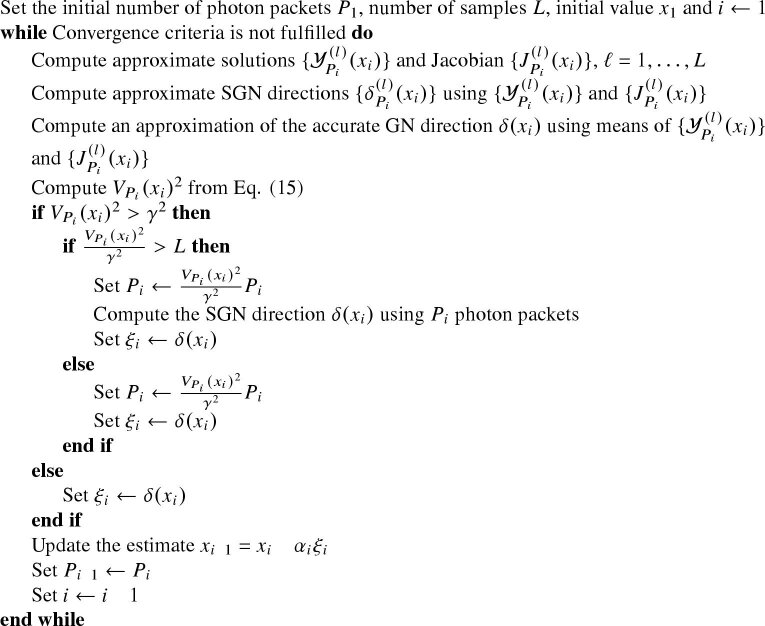

**Fig. 2.**
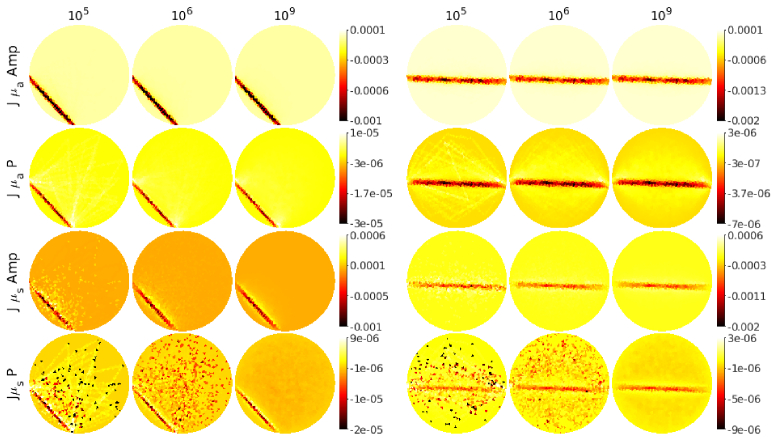
Illustrations of the sensitivity profiles (rows of the Jacobian matrix) for amplitude and phase data with respect to the absorption $\left(J_{\mu_{a}}Amp\;\text{and}\;J_{\mu_{a}}P\right)$ and scattering $\left(J_{\mu_{s}}Amp\;\text{and}\;J_{\mu_{s}}P\right)$ . Two source-detector pairs (columns 1-3 and columns 3-4) were simulated using $10^{5}$ , $10^{6}$ and $10^{9}$ photon packets. The color scale of the images presenting sensitivity profiles of the phase data with respect to the scattering $J_{\mu_{s}}P$ simulated with $10^{5}$ photon packets was cut for visualization since it included some extreme values.

### 3.1 Data simulation

**Fig. 3.**
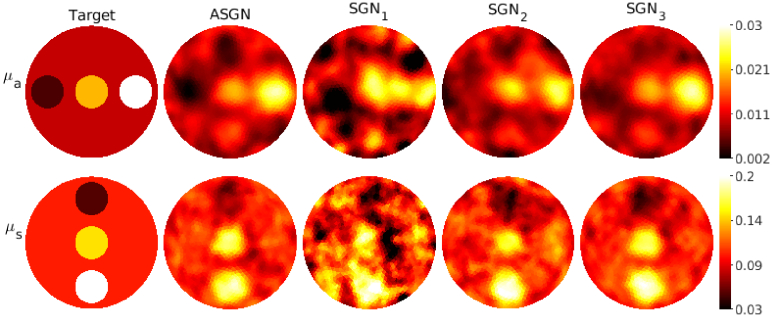
Absorption coefficient $\mu_{a}\;({mm}^{-1})$ (first row) and scattering coefficient $\mu_{s}\;({mm}^{-1})$ (second row) of the target with circular inclusions. Columns from left to right: target parameters (first column), reconstructions obtained using the adaptive stochastic Gauss-Newton method (ASGN) (second column), and reconstructions obtained using stochastic Gauss-Newton method using $10^{5}$ photon packets $\left({SGN}_{1}\right)$ , 10^6^ photon packets $\left({SGN}_{2}\right)$ and $10^{9}$ photon packets $\left({SGN}_{3}\right)$ (columns 3-4 respectively). The total number of photon packets used for one source was $P_{tot}=2.1\cdot 10^{7}$ for the ASGN, $P_{tot}=10^{6}$ for the ${SGN}_{1}$ , $P_{tot}=10^{7}$ for the ${SGN}_{2}$ , and $P_{tot}=10^{10}$ for the ${SGN}_{3}$ . The color scale of the ${SGN}_{1}$ reconstructions is cut so that it is presented in the same scale as the other results.

**Fig. 4.**
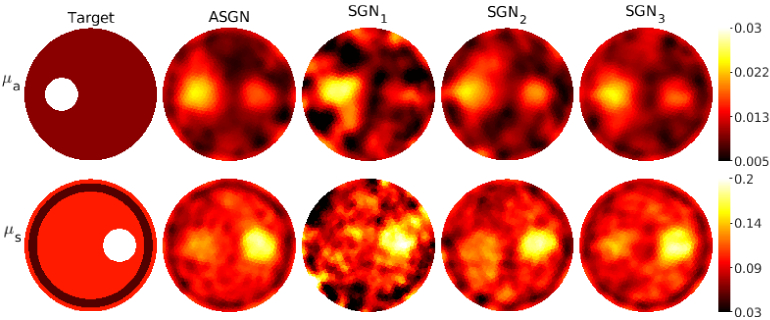
Absorption coefficient $\mu_{a}\;({mm}^{-1})$ (first row) and scattering coefficient $\mu_{s}\;({mm}^{-1})$ (second row) of the target with a low-scattering layer. Columns from left to right: target parameters (first column), reconstructions obtained using the adaptive stochastic Gauss-Newton method (ASGN) (second column), and reconstructions obtained using stochastic Gauss-Newton method using $10^{5}$ photon packets $\left({SGN}_{1}\right)$ , $10^{6}$ photon packets $\left({SGN}_{2}\right)$ and $10^{9}$ photon packets $\left({SGN}_{3}\right)$ (columns 3-4 respectively). The total number of photon packets used for one source was $P_{tot}=1.8\cdot 10^{7}$ for the ASGN, $P_{tot}=10^{6}$ for the ${SGN}_{1}$ , $P_{tot}=10^{7}$ for the ${SGN}_{2}$ , and $P_{tot}=10^{10}$ for the ${SGN}_{3}$ . The color scale of the ${SGN}_{1}$ reconstructions is cut so that it is presented in the same scale as the other results.

**Fig. 5.**
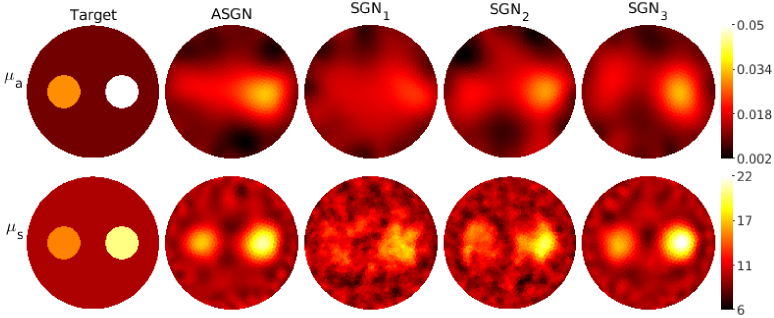
Absorption coefficient $\mu_{a}\;({mm}^{-1})$ (first row) and scattering coefficient $\mu_{s}\;({mm}^{-1})$ (second row) of the highly scattering target. Columns from left to right: target parameters (first column), reconstructions obtained using the adaptive stochastic Gauss-Newton method (ASGN) (second column), and reconstructions obtained using stochastic Gauss-Newton method using $10^{5}$ photon packets $\left({SGN}_{1}\right)$ , $10^{6}$ photon packets $\left({SGN}_{2}\right)$ and $10^{9}$ photon packets $\left({SGN}_{3}\right)$ (columns 3-4 respectively). The total number of photon packets used for one source was $P_{tot}=3.2\cdot 10^{9}$ for the ASGN, $P_{tot}=10^{6}$ for the ${SGN}_{1}$ , $P_{tot}=10^{7}$ for the ${SGN}_{2}$ , and $P_{tot}=10^{10}$ for the ${SGN}_{3}$ .

Three different targets were studied. The targets and their absorption and scattering values are illustrated in Fig. 1 b) – d) and shown later in Figs. 3–5 of Sec. 3.2, where also the results of the reconstructions are shown. The first two targets were low-scattering, and thus they represent the transport regime where the diffusion approximation is not valid. In the third simulation, a higher scattering was considered. The first target consisted of three circular absorbing and three circular scattering inclusions, the second target had an absorbing inclusion and a scattering inclusion inside a low-scattering layer, and the third target had two absorbing and scattering inclusions in a highly scattering medium. In all simulations, the scattering anisotropy parameter was g=0.9 , the refractive index was n=1 , and the frequency of the intensity modulated light was f=100MHz .

To simulate the data, the domain was discretized and the absorption and scattering were expressed in piece-wise constant triangular discretizations consisting of 4574 elements and 2368 nodes (target with circular inclusions), 5506 elements and 2834 nodes (target with a low-scattering layer), and 5844 elements and 3003 nodes (highly scattering target). The data was simulated using the Monte Carlo method as described in Sec. 2.1 using $10^{10}$ photon packets at each source for the low-scattering targets and $10^{9}$ photon packets at each source for the highly scattering target. All source-detector combinations were used in simulations, leading to a simulated data of 256 complex-valued measurements. Amplitude and phase of the exitance were used as the data types of the complex exitance. Gaussian random noise with a zero mean and standard deviation corresponding to 1% of the relative amplitude of the noiseless data was added to the simulated data.

### 3.2 Reconstructions

Absorption and scattering coefficients $x={\left(\mu_{a}\;,\;\mu_{s}\right)}^{T}$ were reconstructed by minimizing (1) in a piece-wise constant discretization using a triangular mesh consisting of 2752 elements and 1457 nodes. Thus, 2752 absorption and 2752 scattering parameters were estimated using 256 simulated measurements. The noise was modeled as a zero mean and uncorrelated, which corresponds to a typical noise model utilized in OT, with the standard deviation $\sigma_{e}$ of 1% of the relative amplitude of the simulated data. This results a diagonal noise covariance matrix and $L_{e}=diag(\sigma_{e,1}^{-1},\ldots ,\sigma_{e,M}^{-1})$ . As a prior model, we used the Ornstein-Uhlenbeck prior that belongs to the class of Matérn covariance functions [37]. In the authors’ previous studies, the prior has been found efficient and versatile for different imaged targets. Ornstein-Uhlenbeck prior is a Gaussian distribution with a covariance matrix defined as

(17) $$\Gamma_{x}(i,j)=\sigma_{x}^{2}\exp\left(-\left\|r_{i}-r_{j}\right\|/\tau \right),$$

where *i* and *j* are the matrix row and column indices, $\sigma_{x}$ is the standard deviation of the prior distribution, $r_{i}$ and $r_{j}$ are the coordinates of the discretization elements, and *τ* is the characteristic length scale parameter that controls the spatial smoothness of the solution. In the simulations, the mean of the prior was set to be the background value of the true target distributions both for absorption and scattering. Further, the standard deviation was set to the third of the difference between the background value and the maximum value of the true target distributions. The values of the prior mean and standard deviation used in simulations are given in Table 1. The characteristic length scale was τ=0.5mm that corresponds to the radius of the circular inclusions.

**Table 1.** Absorption prior mean $\eta_{\mu_{a}}\;({mm}^{-1})$ and standard deviation $\sigma_{\mu_{a}}\;({mm}^{-1})$ and scattering prior mean $\eta_{\mu_{s}}\;({mm}^{-1})$ and standard deviation $\sigma_{\mu_{s}}\;({mm}^{-1})$ used in the simulations.

	$\eta_{\mu_{a}}$	$\sigma_{\mu_{a}}$	$\eta_{\mu_{s}}$	$\sigma_{\mu_{s}}$
Target with circular inclusions	0.01	0.0067	0.1	0.033
Target with a low-scattering layer	0.01	0.0067	0.1	0.033
Highly scattering target	0.01	0.013	10	3.3

Absorption and scattering distributions were reconstructed using the SGN method as described in Sec. 2.2. In order to improve the numerics and to enable the evaluation of the norm test in the simultaneous estimation of absorption and scattering, the absorption and scattering coefficients were scaled using a change of variables [38]

(18) $${\tilde{\mu }}_{a}=\frac{\mu_{a}}{\eta_{\mu_{a}}},\;{\tilde{\mu }}_{s}=\frac{\mu_{s}}{\eta_{\mu_{s}}},$$

where $\eta_{\mu_{a}}$ and $\eta_{\mu_{s}}$ are the (prior) means of absorption and scattering coefficients, respectively. In the SGN algorithm, the Jacobian and prior were scaled respectively. The initial value of the Gauss-Newton algorithm $x_{1}$ was set to the mean of the scaled prior in all simulations. The step length parameter was kept as constant $\alpha_{i}=1$ in all simulations.

The ASGN approach, where the number of photon packets was controlled as described in Sec. 2.3, was compared to the SGN approach where the number of photon packets was kept fixed during the Gauss-Newton iterations. In the ASGN method (Algorithm 1), a sample size L=10 was used. In the norm test, the threshold parameter was γ=1 , and the initial number of photon packets was $P_{1}=1000$ for each source for the first two targets. For the highly scattering target, the threshold parameter was γ=0.6 , and the initial number of photon packets was $P_{1}=10^{4}$ for each source. In the SGN method, different fixed numbers of photon packets were tested: $10^{5},10^{6}$ , and $10^{9}$ (for each source and each iteration). All reconstructions were calculated using 10 iterations. The number of iterations was chosen such that it provided a sufficient convergence for all other simulations except the one with the lowest number of photon packets.

Illustrations of the sensitivity profiles (rows of Jacobians) of two source-detector pairs are shown in Fig. 2 for a different number of photon packets. The sensitivity profiles were computed using absorption and scattering values $\mu_{a}=0.01\;{mm}^{-1}$ and $\mu_{s}=0.1\;{mm}^{-1}$ . As it can be seen, the stochastic noise affects the sensitivity profiles especially when a low number of photon packets is used. This is especially evident in the Jacobian for scattering.

Reconstructed absolute absorption and scattering parameters for the target with circular inclusions are shown in Fig. 3, for the target with a low-scattering layer in Fig. 4, and for the highly scattering target in Fig. 5 using both the adaptive approach and the different fixed numbers of photon packets. As it can be seen, the adaptive approach provides absorption and scattering reconstructions that highly resemble the reconstructions obtained using a high number of photon packets $10^{9}$ . The locations of the inclusions and the low-scattering layer can be distinguished using both of these methods, and almost the same contrast in the parameter values is reached when compared to the true targets in the targets in the transport regime for both absorption and scattering. In the highly scattering case, however, the absorption contrast is weak. This can be due to the scattering being so much larger than the absorption, and the reconstructions could be improved, for example, by using different scaled data types, which have previously been utilized in diffuse OT [13,38], improving prior information, and adjusting the SGN parameters such as the norm test criteria and number of photon packets. The reconstructions calculated using a low fixed number of photon packets, $10^{5}$ and $10^{6}$ , look noisy and the locations of the inclusions are not as clearly distinguished as with the adaptive approach and with the the approach using $10^{9}$ photon packets.

**Fig. 6.**
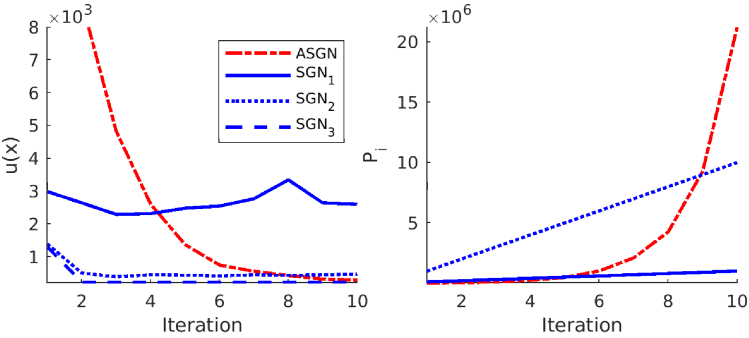
Left image: Minimized functional u(x) against the iteration for the target with circular inclusions in the adaptive stochastic Gauss-Newton method (ASGN) and in the stochastic Gauss-Newton method where a fixed number of $10^{5}$ photon packets $\left({SGN}_{1}\right)$ , $10^{6}$ photon packets $\left({SGN}_{2}\right)$ and $10^{9}$ photon packets $\left({SGN}_{3}\right)$ were used. Right image: Cumulative sum of the used photon packets $P_{i}$ against the iteration. The cumulative sum of the photon packets used in the stochastic Gauss-Newton method, where a fixed number of $10^{9}$ photon packets $\left({SGN}_{3}\right)$ was used, is larger than the image axis and was therefore not plotted in the right image.

**Fig. 7.**
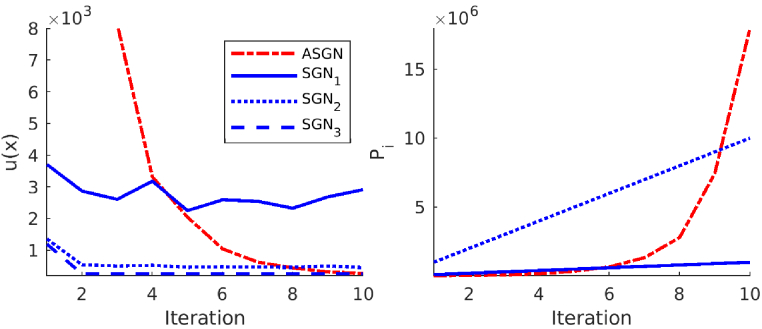
Left image: Minimized functional u(x) against the iteration for the target with a low-scattering layer in the adaptive stochastic Gauss-Newton method (ASGN) and in the stochastic Gauss-Newton method where a fixed number of $10^{5}$ photon packets $\left({SGN}_{1}\right)$ , $10^{6}$ photon packets $\left({SGN}_{2}\right)$ and $10^{9}$ photon packets $\left({SGN}_{3}\right)$ were used. Right image: Cumulative sum of the used photon packets $P_{i}$ against the iteration. The cumulative sum of the photon packets used in the stochastic Gauss-Newton method, where a fixed number of $10^{9}$ photon packets $\left({SGN}_{3}\right)$ was used, is larger than the image axis and was therefore not plotted in the right image.

**Fig. 8.**
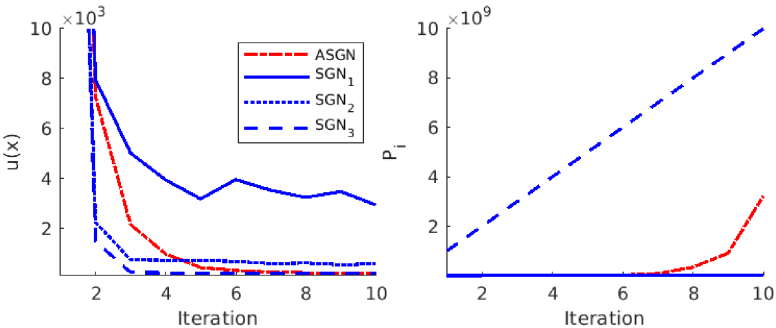
Left image: Minimized functional u(x) against the iteration for the highly scattering target in the adaptive stochastic Gauss-Newton method (ASGN) and in the stochastic Gauss-Newton method where a fixed number of $10^{5}$ photon packets $\left({SGN}_{1}\right)$ , $10^{6}$ photon packets $\left({SGN}_{2}\right)$ and $10^{9}$ photon packets $\left({SGN}_{3}\right)$ were used. Right image: Cumulative sum of the used photon packets $P_{i}$ against the iteration.

The value of the minimized functional u(x) against the iteration and a cumulative sum of used photon packets for one source against the iteration are shown in Fig. 6 for the target with circular inclusions, in Fig. 7 for the target with a low-scattering layer, and in Fig. 8 for the highly scattering target. As it can be seen from the left images of Figs. 6–8, the adaptive approach converges slower than the approaches that use a larger number of photon packets. On the other hand, comparing the right images of Figs. 6–8 show that the number of photon packets used in the ASGN during the first iterations is low, resulting in significant savings in the total number of used photon packets, which can be considered as savings in computation resources. The total number of used photon packets for one source in the ASGN method for the target with circular inclusions was $P_{tot}=2.1\cdot 10^{7}$ , for the target with a low scattering layer $P_{tot}=1.8\cdot 10^{7}$ , and for the highly scattering target $P_{tot}=1.8\cdot 10^{9}$ . Looking at the left images of Figs. 6–8 also shows that using a low number of photon packets in the SGN method, does not provide as good convergence as the adaptive approach. This is due to the stochastic noise that is present in Monte Carlo simulations and limits the convergence of the minimization algorithm. Loosely speaking, one can interpret the behavior of the algorithm such that the stochastic noise causes ’jumping’ of the estimated parameters in the vicinity of the true minimum.

### 3.3 Study on the randomness of the methodology and statistics

**Fig. 9.**
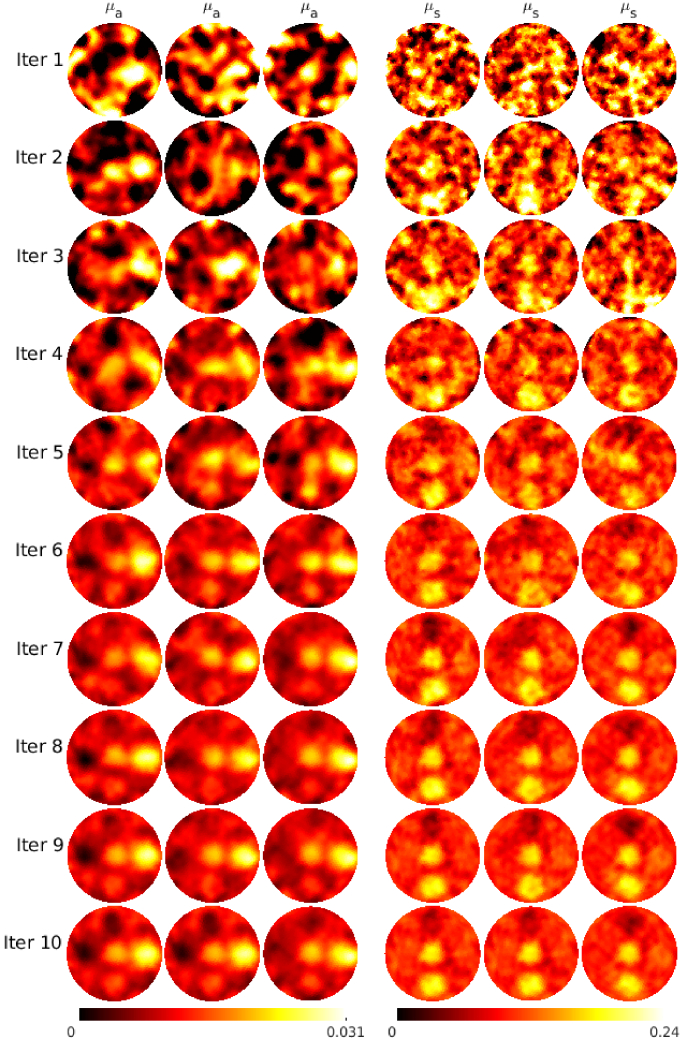
Progress of the absorption $\mu_{a}\;({mm}^{-1})$ (columns 1-3) and scattering $\mu_{s}\;({mm}^{-1})$ (columns 4-6) reconstructions on iterations 1 to 10 of the adaptive stochastic Gauss-Newton algorithm for the target with circular inclusions for three repetitive reconstructions with a sample size L=10 . The color scales of the reconstructions on iterations 1 and 2 are cut so that they are presented in the same scale as the other iterations.

To study the randomness of the adaptive SGN methodology, image reconstruction of the target with circular inclusions was repeated multiple times and studied also using different sample sizes *L*. The reconstructed absorption and scattering on different iterations are shown in Fig. 9 for three different simulations with a fixed sample size L=10 . As it can be seen, as the number of photon packets is lower in the early iterations, the absorption and scattering of different simulations look different. Then, as the iteration proceeds and the minimized functional decreases, the absorption and scattering begin to resemble each other, eventually leading to similar final reconstructions.

**Fig. 10.**
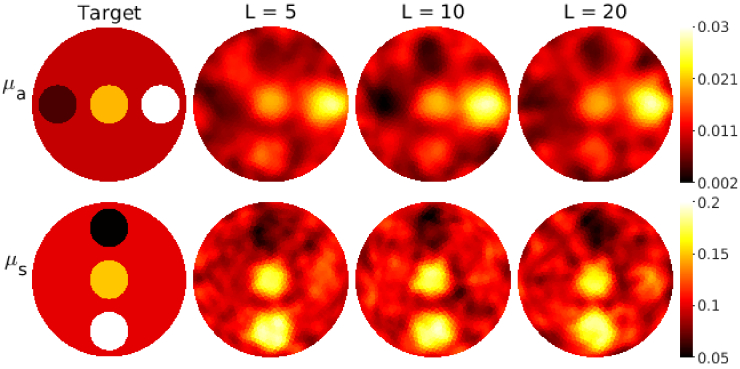
Absorption coefficient $\mu_{a}\;({mm}^{-1})$ (first row) and scattering coefficient $\mu_{s}\;({mm}^{-1})$ (second row) of the target with circular inclusions. Columns from left to right: target parameters (first column), reconstructions obtained using the adaptive stochastic Gauss-Newton method (ASGN) with the sample size L=5 (second column), L=10 (third column), and L=20 (fourth column). For the sample size L=5 , reconstructions after 20 iterations are shown, and for the sample size L=10 and L=20 , reconstructions after 10 iterations are shown.

**Fig. 11.**
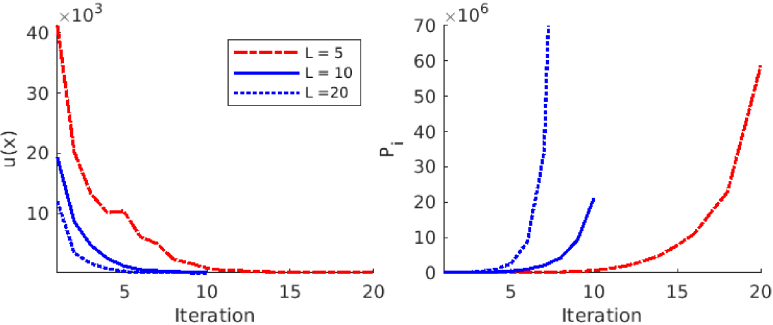
Left image: Minimized functional u(x) against the iteration for the target with circular inclusions in the adaptive stochastic Gauss-Newton method (ASGN) with the sample size L=5,10,and 20 . Right image: Cumulative sum of the used photon packets $P_{i}$ against the iteration. The cumulative sum of the photon packets used with the sample size L=20 on iterations 7-10 is larger than the image axis and was therefore not plotted in the right image.

The absorption and scattering reconstructed using different sample size L=5,10,and 20 are shown in Fig. 10. Further, the minimized functional u(x) and the cumulative number of photon packets $P_{i}$ against the iteration are shown in Fig. 11. As it can be seen, the minimization problem converges faster if a larger sample size is used. As a consequence, the sample size L=5 required more than 10 iterations to converge and the sample size L=20 required less than 10 iterations. On the other hand, the use of a larger sample size increases the number of photon packets on each iteration more than a smaller sample size. In the end, all simulations provided similar reconstructions both for absorption and scattering.

Since the SGN is a stochastic method, it may provide different results when a reconstruction is calculated using a different set of photon packet realizations. Therefore, in order to provide statistical information of the method, reconstructions of the target with circular inclusions and the target with a low-scattering layer were repeated 10 times using the ASGN method and the SGN method with a fixed number of photon packets $10^{5}$ and $10^{6}$ . These results were used to calculate the means and standard deviations of the minimized functional u(x) , relative errors of the absorption $E_{\mu_{a}}$ and scattering $E_{\mu_{s}}$ estimates, and the total number of photon packets $P_{tot}$ at the end of the iteration for one source. The relative errors were calculated from

(19) $$E_{\mu_{a}}=\frac{\left\|{\hat{\mu }}_{a}-\mu_{a,true}\right\|}{\left\|\mu_{a,true}\right\|}\cdot 100\%,\;E_{\mu_{s}}=\frac{\left\|{\hat{\mu }}_{s}-\mu_{s,true}\right\|}{\left\|\mu_{s,true}\right\|}\cdot 100\%$$

where ${\hat{\mu }}_{a}$ and ${\hat{\mu }}_{s}$ are the estimated and $\mu_{a,true}$ and $\mu_{s,true}$ are the (true) simulated absorption and scattering coefficients, respectively, and the norm is the Euclidean norm. The results obtained using $10^{9}$ were considered as a reference. These reference values, for the target with circular inclusions, are: the end value of the minimized functional u(x)=231 , the relative error of absorption $E_{\mu_{a}}=23.3\;\%$ , the relative error of scattering $E_{\mu_{s}}=12.8\;\%$ , and the total number of photon packets $P_{tot}=10^{10}$ . For the target with a low-scattering layer: the end value of the minimized functional u(x)=260 , the relative error of absorption $E_{\mu_{a}}=27.9\;\%$ , the relative error of scattering $E_{\mu_{s}}=17.5\;\%$ , and the total number of photon packets $P_{tot}=10^{10}$ .

**Fig. 12.**
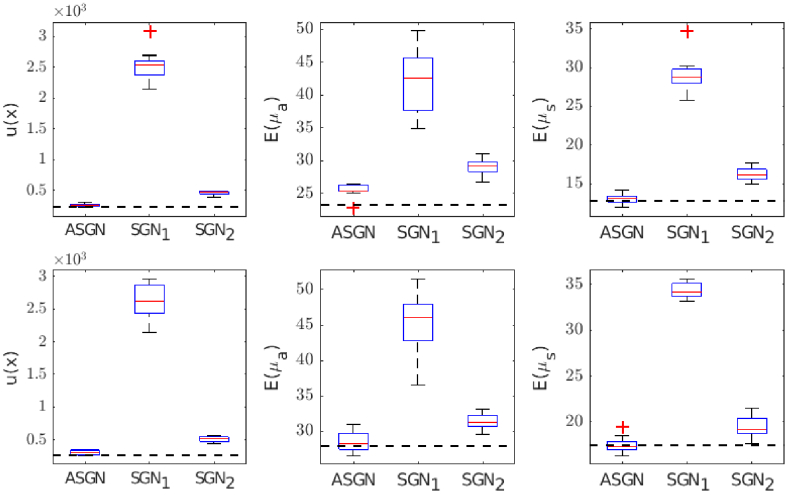
Value of the minimization functional u(x) (first column) and the relative errors of the absorption $E_{\mu_{a}}$ (second column) and scattering $E_{\mu_{s}}$ (third column) estimates calculated using the adaptive stochastic Gauss-Newton method (ASGN) and the stochastic Gauss-Newton method with a fixed number of photon packets $10^{5}\;({SGN}_{1})$ and $10^{6}\;({SGN}_{2})$ and repeated 10 times. The target with circular inclusions (first row) and the target with a low-scattering layer (second row). The median of the values is marked as a red horizontal line, the 20th and 75th percentile is included in a blue box and the whiskers (black vertical lines) include all samples excluding the outliers that are marked with a plus symbol. In addition, the values of the reference estimate obtained using the stochastic Gauss-Newton method with a fixed number of photon packets $10^{9}\;({SGN}_{3})$ are marked with a black dashed line.

The calculated mean and standard deviations of the minimized functional u(x) , relative error of absorption $E_{\mu_{a}}$ , relative error of scattering $E_{\mu_{s}}$ and total number of photon packets $P_{tot}$ are presented in Table 2 for the ASGN method and SGN method with the fixed number of photon packets $10^{5}$ and $10^{6}$ . Furthermore, the values of the minimized functional u(x) , and the relative errors of the absorption and scattering coefficients are visualized in Fig. 12. As it can be seen, the adaptive approach converges closest to the reference solution providing the lowest values of the minimized functional and relative errors, when compared to the approaches with the fixed number of photon packets. Furthermore, it can be seen that, as the number of used photon packets increases, variation in the values of the minimized functional and relative errors decrease.

**Table 2.** Mean and standard deviations of the minimized functional u(x) , relative errors of absorption $E_{\mu_{a}}$ (%) and scattering $E_{\mu_{s}}$ (%) estimates and total number of photon packets $P_{tot}$ for one source. The reconstructions were calculated using the adaptive stochastic Gauss-Newton method (ASGN) and the stochastic Gauss-Newton method with a fixed number of photon packets $10^{5}\;({SGN}_{1})$ and $10^{6}\;({SGN}_{2})$ and repeated 10 times.

	Target with circular inclusions			
	
	u(x)	$E_{\mu_{a}}$	$E_{\mu_{s}}$	$P_{tot}$
ASGN	259±27	25.3±1.0	13.1±0.6	$(2.4\pm 0.9)\cdot 10^{7}$
${SGN}_{1}$	2524±263	42.1±5.0	29.1±2.3	$1\cdot 10^{6}$
${SGN}_{2}$	463±31	29.0±1.2	16.3±0.9	$1\cdot 10^{7}$
	Target with a low-scattering layer			
	
	u(x)	$E_{\mu_{a}}$	$E_{\mu_{s}}$	$P_{tot}$
	
ASGN	301±33	28.6±1.4	17.5±1.0	$(1.5\pm 0.8)\cdot 10^{7}$
${SGN}_{1}$	2594±283	45.3±4.2	34.4±0.9	$1\cdot 10^{6}$
${SGN}_{2}$	509±42	31.3±1.2	19.5±1.3	$1\cdot 10^{7}$

## 4. Discussion and conclusions

In this work, utilizing the Monte Carlo method in the absolute imaging problem of OT was considered. The Monte Carlo simulation was based on the photon packet method. The image reconstruction problem was formulated as a minimization problem that was solved using the SGN method [27,29,30]. In the construction of the Jacobian matrix for scattering, the perturbation approximation was utilized [22,28]. The SGN method was studied using an adaptive approach, where the number of photon packets was controlled during iterations by studying the variance of the minimization direction by a norm test, and with different fixed numbers of photon packets.

The results show that the Monte Carlo method can be utilized in the absolute imaging problem of OT. It was also shown that the number of the photon packets affects on the performance of the method. Using a low number of photon packets can provide noisy-looking images with large relative errors when compared to the true absorption and scattering values. On the other hand, using a large number of photon packets is computationally expensive. Therefore, the adaptive approach, where the number of photon packets is controlled during the iteration, can provide an efficient approach to the OT image reconstruction with Monte Carlo. However, the performance of the methodology depends on the chosen sample size and the threshold parameter of the norm test, and thus more research is needed for their optimal choice.

The future work includes extension of the methodology to three dimensions and consideration of computationally more efficient solutions. For example in [27], an adjoint Monte Carlo and a gradient based optimization method, that does not require constructing the Jacobian matrix, were utilized in quantitative photoacoustic tomography. However, the method was utilized only in estimating the absorption, which is only a mildly ill-posed problem in quantitative photoacoustic tomography.

To conclude, it was shown that the Monte Carlo method for light transport can be utilized in estimating absorption and scattering in OT. The method can be utilized in situations where the diffusion approximation is not valid. Furthermore, it was shown that an adaptive control of the photon packets during the iterations can lead to efficient convergence of the image reconstruction problem, with reasonable computational resources.

## Data Availability

Data underlying the results presented in this paper are not publicly available at this time but may be obtained from the authors upon reasonable request.
